# Retraction: *In vivo* clearance of apoptotic debris from tumor xenografts exposed to chemically modified tetrac: is there a role for thyroid hormone analogues in efferocytosis?

**DOI:** 10.3389/fendo.2024.1480220

**Published:** 2024-08-21

**Authors:** Frontiers Editorial Office

Following publication, concerns were raised regarding the integrity of the images in the published figures. Image duplication concerns were identified in Figure 2 and 3.

The authors failed to provide a satisfactory explanation during the investigation, which was conducted in accordance with Frontiers’ policies. As a result, the data and conclusions of the article have been deemed unreliable and the article has been retracted. The author(s) did not provide a response to this Retraction.

